# How Senses Work Together: Cross-Modal Interactions between Primary Sensory Cortices

**DOI:** 10.1155/2018/5380921

**Published:** 2018-12-17

**Authors:** Manuel Teichert, Jürgen Bolz

**Affiliations:** Institute of General Zoology and Animal Physiology, University of Jena, 07743 Jena, Germany

## Abstract

On our way through a town, the things we see can make us change the way we go. The things that we hear can make us stop or walk on, or the things we feel can cause us to wear a warm jacket or just a t-shirt. All these behaviors are mediated by highly complex processing mechanisms in our brain and reflect responses to many important sensory inputs. The mammalian cerebral cortex, which processes the sensory information, consists of largely specialized sensory areas mainly receiving information from their corresponding sensory modalities. The first cortical regions receiving the input from the outer world are the so called primary sensory cortices. Strikingly, there is convincing evidence that primary sensory cortices do not work in isolation but are substantially affected by other sensory modalities. Here, we will review previous and current literature on this cross-modal interplay.

## 1. Introduction

In their natural environment, humans and animals receive multimodal sensory stimuli. The sensation of these external stimuli already begins at the level of specialized sensory organs like the eyes, the ears, or the skin. From here, the sensory information is relayed to certain structures of the central nervous system before it enters the cortex of the brain. The first cortical regions which receive the input from the outer world are the primary sensory cortices, such as the primary visual cortex (V1), the primary auditory cortex (A1), or the primary somatosensory cortex (S1). Generally, these regions are anatomically separated. However, some previous studies in humans and experimental animals challenged the traditional view that these cortices only process information coming from their corresponding sensory organs and that multisensory integration only exists in “higher-order” cortices [1–6]. Rather, there is now convincing evidence for a multimodal interplay already at the level of the primary sensory cortices [7–9].

## 2. Connectivity between Primary Sensory Cortices

Several studies provided convincing evidence that primary sensory cortices are anatomically interconnected. For instance, anatomical tracing studies in rodents revealed relatively strong direct corticocortical projections from A1 and S1 to V1 [1, 7, 9–11]. Conversely, V1 was shown to project predominantly to S1 and only weakly to A1, whereas S1 sends moderate projections to A1 and receives projections from there [1]. These findings clearly demonstrate a high anatomical connectivity between the primary sensory cortices which is, however, often asymmetric.

Such anatomical connections suggest the presence of a functional multimodal interplay between these early sensory cortical regions. This idea is supported by previous findings demonstrating that primary sensory cortices receive subthreshold inputs originating from other sensory modalities [8–10, 12–15]. Already more than 50 years ago, several studies found that neurons in the cat visual cortex (areas 17, 18, and 19) did respond not only to visual but also to auditory stimuli [16–18] suggesting a multimodal integration in early sensory cortices. Later on, it was shown that many V1 neurons in rodents also respond to somatosensory and auditory stimuli [8, 15]. Specifically, both stimulation of the vibrissae on the snout (whiskers) of mice and auditory stimulation evoked hyperpolarization in V1 [8]. Conversely, there is evidence that visual stimulation can depolarize layer 2/3 neurons in S1 and layer 6 neurons in A1 [8, 19]. These findings obtained in rodents suggest that the neuronal activity of neurons in early sensory cortices can be modulated by inputs coming from other sensory modalities. Moreover, this idea is also supported by findings made in primates [13, 20], ferrets [21], and cats [22] indicating that cross-modal integration of primary sensory cortices is widely conserved across mammalian species. Recently, the effects of auditory stimulation on visual processing have received great attention [8, 9, 23–25]. In the following paragraphs, we will first review the cross-modal interplay between audition and vision at the level of primary sensory cortices, and in the second part, we will discuss cross-modal interactions between somatosensation and vision. Our focus will be on alterations after sensory deprivation or loss in adult animals on the remaining intact sense at the systemic and behavioral level.

## 3. Functional Interplay between Primary Sensory Cortices

Iurilli and colleagues [8] found that salient activations of A1 due to sound stimulation or optogenetic stimulation hyperpolarized supragranular pyramidal cells in V1. Moreover, a cortical transection between A1 and V1 abolished these sound-induced responses in V1 [8, 23]. These results indicate that V1 receives suppressive inputs from the activated auditory cortex, most likely via direct corticocortical connections described above. By addressing a similar issue, a recent study could demonstrate by in vitro electrophysiological recordings that layer 1 and layer 2/3 inhibitory neurons in V1 receive direct excitatory input from the auditory cortex [9]. Taken together, these results suggest that sound-evoked hyperpolarization in layer 2/3 excitatory cells in V1 is mediated by sound-driven activations of V1 inhibitory neurons. Although these electrophysiological studies provide evidence for a functional interplay between A1 and V1, it remained unclear, however, how this interplay is reflected at the systemic level of these cortices.

**Figure 1:**
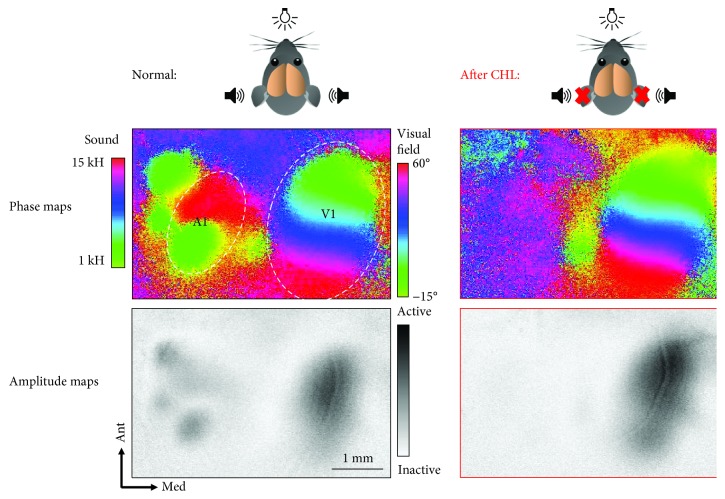
Simultaneous intrinsic signal imaging of the primary auditory (A1) and primary visual cortex (V1) before and after the induction of a conductive hearing loss (CHL). (Upper row) tonotopically organized phase map of the auditory cortex and visuotopically organized phase map of the visual cortex obtained before and after CHL in the same animal. Note that after CHL, the map of the auditory cortex was absent. (Lower row) corresponding amplitude maps. Generally, darker activity patches indicate higher sensory-evoked activity. After CHL, visually evoked V1 activity substantially increased. Thus, in normal mice, auditory stimulation suppresses visually elicited V1 responses.

To address the issue how V1 responsiveness is influenced by auditory stimulation at the systemic level, we recently developed a novel method to simultaneously record a visuotopic map in V1 and a tonotopic map in A1 in mice using Fourier-based intrinsic signal imaging [7] (Figure 1). For this, animals were simultaneously stimulated with a sound sweep ranging from 1 to 15 kHz at 70 dB and a visual stimulus, a moving white light bar on a black background with a horizontal orientation. In normal mice, this bimodal sensory stimulation evoked high neuronal activity in A1 and V1. However, in experimental animals with conductive hearing loss (CHL), induced by bilateral removal of the malleus, there was practically no response to the 70 dB sound stimuli in A1. Strikingly, however, there was an immediate increase by about 15% in V1 responses to the visual stimulus presented simultaneously with the auditory stimulus. [7, 26] (Figure 1, lower row). Thus, these results suggest that a high activation of the auditory cortex suppresses visually evoked V1 activity. However, when A1 activity decreases (like after CHL), the break on visually elicited V1 activity is released leading to a concomitant increase in V1 activation. In accordance with this finding, Iurilli and colleagues [8] could demonstrate by electrophysiological recordings that when A1 activity was low, like in the absence of sounds, neuronal activity in V1 increased. Taken together, these results indicate that sounds act to suppress visually evoked activity in V1.

In apparent contrast, a previous study demonstrated that visually driven activity of V1 neurons was stronger under concurrent auditory stimulation, only if the visual stimulus was presented in the preferred orientation [9]. However, visual stimulation with a nonpreferred orientation together with sounds decreased sensory-evoked activity of V1 neurons. Thus, averaged across all presented orientations, neuronal firing was weaker under bimodal stimulation compared to visual stimulation alone [24]. Since we used a visual stimulus with only one orientation, the results reported by Ibrahim and colleagues [9] are in line with the results of our study [7]. Specifically, intrinsic signal imaging does not allow the measurement of orientation tuning of single V1 neurons because orientation preference throughout V1 is organized in a “salt and pepper” manner [27, 28]. Hence, we cannot make statements about whether cells which preferred the orientation of the presented light bar (horizontal) responded stronger under combined visual and auditory stimulation. However, in agreement with the results obtained by Ibrahim et al. [9], we hypothesize that cells with a preferred orientation tuning different from horizontal, likely the majority of V1 neurons, have a weaker visually evoked V1 activity when a sound is delivered simultaneously. Taken together, one could argue that the effects of sounds on V1 activity might differ depending on the visual stimulus applied simultaneously and on the neuronal characteristics observed.

**Figure 2:**
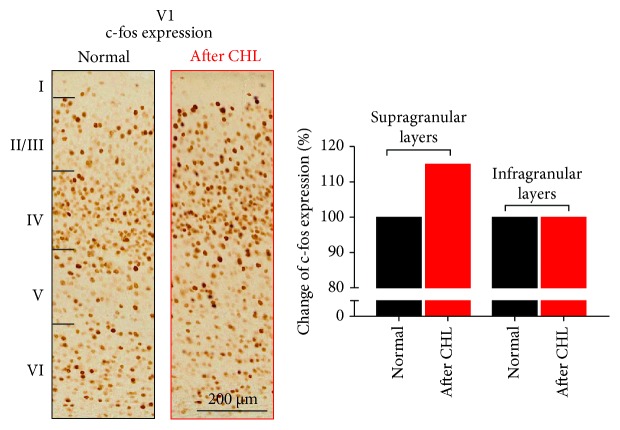
Expression of the neuronal activity marker c-fos in the supragranular layers of V1 was increased after CHL. The number of c-fos-stained cells in V1 was increased in the supragranular layers, but not in the infragranular layers. These data suggest that a CHL causes an increase in neuronal activity at the neuronal level in the upper cortical layers of V1.

To investigate which cortical layers of V1 are responsible for this regulation, we performed immunohistological stainings with the neuronal activity marker *c-fos* [29–31] in V1. In accordance with the imaging results, we found that the number of *c-fos*-positive pyramidal cells in supragranular layers of V1 was increased after CHL, indicating an increased neuronal firing in these cortical layers (Figure 2) [7]. This result is perfectly in line with the finding that sound stimulation predominantly alters neuronal activity of pyramidal cells in the superficial layers of V1, as revealed by electrophysiological recordings [8, 9]. In contrast, we further found that the number of *c-fos-*positive inhibitory neurons in infragranular and supragranular layers of V1 was decreased after CHL. These results suggest that a reduction of the inhibitory tone in V1 (after CHL) is the major reason for the increase in visually evoked activity in this cortex, as revealed by intrinsic signal imaging. Likewise, decreasing GABAergic inhibition in V1 immediately abolished the suppressive effect of sounds and led to an increase in neuronal firing in V1 at the single unit level [8], underlining the importance of inhibition in the cross-modal interaction of A1 and V1.

**Figure 3:**
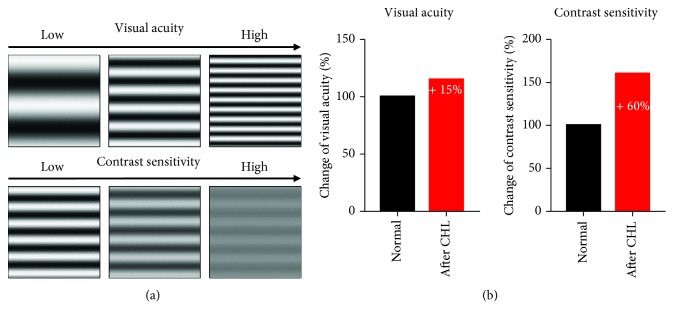
Acute conductive hearing loss (CHL) leads to an immediate improvement of visual acuity and contrast sensitivity as revealed by intrinsic signal imaging in V1. (a) Visual stimuli were sine wave gratings with increasing spatial frequency (upper row) and decreasing contrasts, respectively, (lower row). After CHL, also weak visual stimuli (higher spatial frequency, low contrast) evoked high responses in V1 compared to before CHL conditions. (b) Hence, the determined values of visual acuity and contrast sensitivity were significantly enhanced by 15% and 60%, respectively, after CHL.

The intricate involvement of hearing on V1 processing raises the question of the influence of sounds on visual abilities. Often used benchmarks for the determination of visual abilities are visual acuity, the ability to resolve fine details in the visual scene and contrast sensitivity, and the ability to discriminate between differences in the brightness of visual stimuli as illustrated in Figure 3(a). Thus, in order to investigate whether audition affects vision, we determined visual acuity and contrast sensitivity in mice which were simultaneously stimulated with sounds and visual stimuli before and after CHL. For this, we developed a novel method to determine V1 spatial frequency and contrast tuning using intrinsic signal imaging [32]. Strikingly, we found that CHL immediately leads to marked enhancement of both visual acuity and contrast sensitivity (Figure 3(b)). These results suggest that CHL leads to a rapid improvement of visual abilities. Conversely, a salient activation of A1 evoked by auditory stimulation acts to impair vision [7]. This interpretation is in line with the findings that acoustic stimuli reduced the behavioral response of mice conditioned to a visual stimulus [8, 23] and that in humans visual perception gets impaired under concurrent auditory stimulation [33]. In contrast, as mentioned above, Ibrahim et al. [9] suggested that auditory stimulation sharpens orientation selectivity of V1 neurons. Thus, it has been speculated that this effect might boost visual performance in mice [24]. At the first glance, these results seem to be at variance. However, Ibrahim and colleagues did not investigate whether the effect, obtained with single unit recordings in V1, is also reflected in the behavior or at the systemic level. In addition, orientation selectivity represents only one single aspect of mouse vision. Thus, further studies are required to unravel the precise effects of hearing on the ability to see.

## 4. Cross-Modal Plasticity

There is increasing evidence that the early loss of one sensory modality can lead to compensatory cross-modal improvements of the remaining senses [34, 35]. Such changes are broadly referred to as “cross-modal plasticity” [34, 35]. For example, congenitally deaf individuals and experimental animals display superior visual abilities [36, 37], and blind individuals have enhanced auditory functions [38, 39]. It has been proposed that such improvements appear because the deprived cortex becomes driven by the spared sensory modalities. For instance, human studies have shown that the visual cortex of congenitally blind subjects can be activated during tactile tasks, like Braille reading, or auditory processing [40–42]. Furthermore, in deaf individuals, the auditory cortex was shown to be activated by visual stimuli [43, 44].

Interestingly, the improved function of the remaining senses requires neuronal activity in the deprived cortex [37, 41]. For instance, superior visual abilities in congenitally deaf cats were eliminated by deactivation of several auditory cortical regions [37]. These results suggest that cross-modal recruitment is involved in sharpening the spared senses. Previously, it was thought that such changes are results of long-term adaptions [45] or stabilizations of exuberant corticocortical connections which are initially established during early postnatal development and then retracted at later developmental stages [46, 47]. However, more recently, it was demonstrated that also simply blindfolding adults with normal vision for only a few days can also cause activations of the visual cortex while Braille reading [48]. In summary, these studies highlight the ability of the juvenile and adult deprived cortex that neuronal activity from spared sensory modalities can “hitchhike” the deprived sensory cortices. Such changes are broadly referred to as “cross-modal recruitment” which represents one category of cross-modal plasticity [35].

What might be a potential mechanism for this cross-modal cortical activation? Many studies could demonstrate that sensory deprivation results in homeostatic adjustments recovering the neuronal activity of the deprived cortex. For instance, vison loss induced by blocking the activity of the optic nerve with TTX, dark exposure, binocular enucleation, or laser-induced retinal lesions scale up excitatory synapses of layer 2/3 pyramidal neurons in V1 [49–53]. Moreover, a recent study demonstrated that the similar effects also take place in A1 after auditory deprivation [54]. This mechanism is commonly referred to as “synaptic scaling” [55, 56]. The main feature of homeostatic “synaptic scaling” is that it globally increases (or decreases) the strength of all neuron synapses in a multiplicative manner [55, 57]. Hence, it was proposed that inputs in the deprived primary sensory cortex coming from other sensory modalities [8, 9] also become amplified during homeostatic synaptic adaptions [35]. In general, the precise mechanisms for cross-modal recruitment are still poorly understood. However, homeostatic synaptic plasticity might represent a potential underlying the mechanism for these intriguing cross-modal adaptions of the adult cerebral cortex.

Recent studies extended the view of cross-modal recruitment by demonstrating that the loss of one sense also provokes massive plastic changes in the spared sensory cortices. For instance, it has been shown that only one week of visual deprivation in juvenile mice acts to sharpen the tuning of layer 2/3 cells in the barrel field of S1 [58]. Likewise, other studies showed that the same intervention (visual deprivation) strengthens thalamocortical synapses in the spared A1 in juvenile mice [59]. This effect was accompanied by a refinement of intracortical circuits in A1 [60, 61] and an increased sensitivity and frequency tuning of A1 neurons [59]. This type of cross-modal plasticity is called “compensatory plasticity” [35]. During early life, primary sensory cortices of mammals display a high degree of plasticity [62–64]. Hence, it was thought that cross-modal changes could be attributed to the tremendous potential of juvenile sensory cortices to undergo experience-dependent plasticity. However, it has been demonstrated that one week of dark exposure also induced plastic changes in the spared A1 in adult mice [59]. Thus, these findings suggest that compensatory plasticity in a spared primary sensory cortex is a general feature of the young and adult brain, at least in experimental animals. The studies mentioned above used visual deprivations and then investigated the effects on the remaining primary sensory cortices. However, evidence for the effects of the deprivation of other senses on the visual cortex is rare. Moreover, it remained unclear whether the observed cross-modal changes are also reflected at the level of sensory cortex-dependent behavior. In other words, does the late-onset deprivation of a sensory modality enhance behavioral performance mediated by a spared primary sensory cortex?

**Figure 4:**
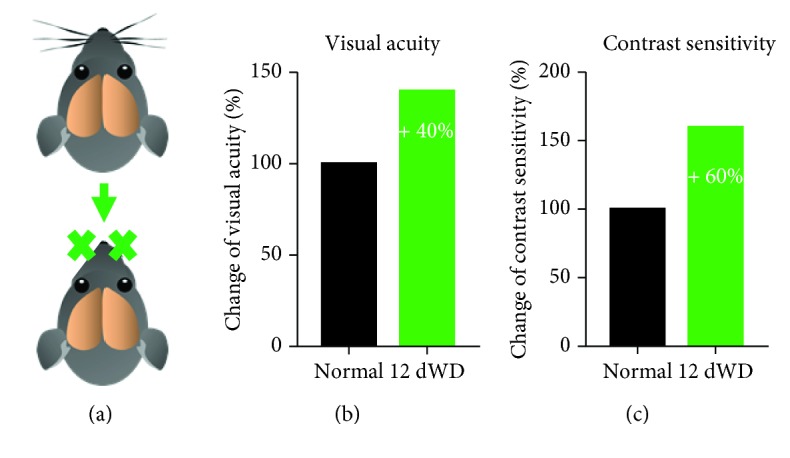
Whisker deprivation (WD) markedly enhances visual acuity and contrast sensitivity as measured in V1 using intrinsic signal imaging. (a) WD was performed by bilaterally removing the macrowhiskers on the snout (b) After WD for 12 days, visual acuity was increased by about 40%. (c) The same treatment led to a massive improvement of contrast sensitivity by 60%.

**Figure 5:**
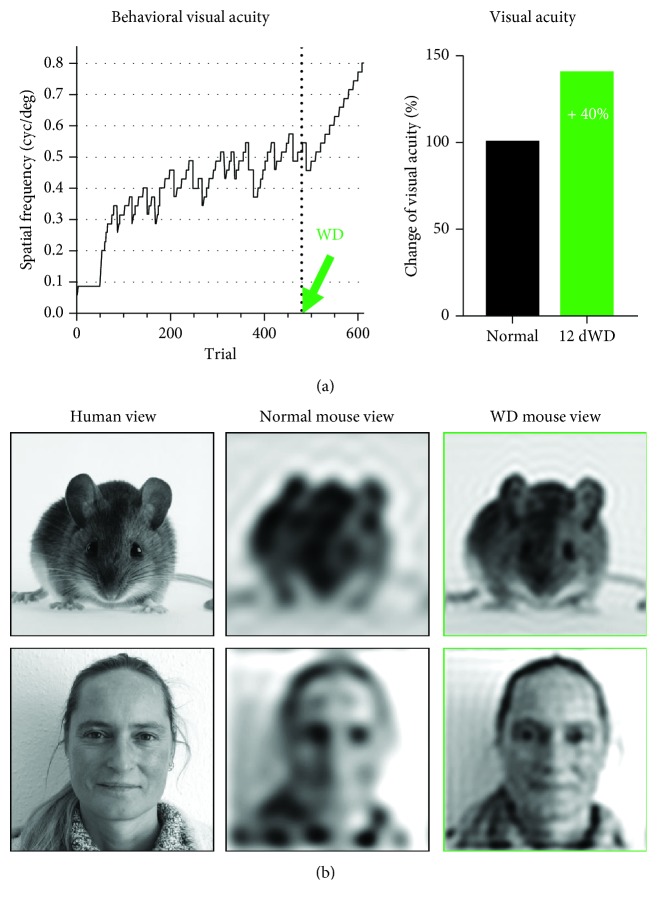
A prolonged whisker deprivation (WD) massively boosts behavioral visual performance. (a) Representative trace of the visual acuity of one WD animal obtained by the behavioral visual water task. Before WD, the animal reached a level of visual acuity (0.5 cycle per degree) which is normal for mice [32, 70]. Strikingly, during some days after WD, behavioral visual acuity dramatically increased to a value higher than 0.7 cycle per degree. Averaged across all mice used, visual acuity was found increased by almost 40%. (b) Illustration of visual improvements in WD mice compared to normal human vision.

In order to address this issue, we investigated the cross-modal effects of a partial somatosensory deprivation lasting 12 days induced by bilateral whisker deprivation (WD) on V1 function and visually mediated behavior in fully adult mice older than 120 days [65] (Figures 4 and 5). For this, we first determined V1 spatial frequency and contrast tuning in control and WD mice using intrinsic signal imaging [32]. Strikingly, we found that V1 responses evoked by weak visual stimuli were massively increased after WD which resulted in a marked improvement of V1 spatial frequency and contrast tuning. Quite remarkably, visual acuity and contrast sensitivity were enhanced by almost 40% and 60%, respectively (Figures 4(b) and 4(c)) [65]. In line with the studies mentioned above [58, 59, 66], these results demonstrate that a short-term deprivation of one sense refines neuronal processing in the spared sensory cortices, even in adult mice. As a next step, we investigated whether these effects are also reflected at the level of visually guided behavior (Figure 5). Behavioral visual acuity and contrast sensitivity were determined using the visual water task, a visual cortex-dependent visual discrimination task based on reinforcement learning [67]. Strikingly, WD also dramatically improved behavioral contrast sensitivity and visual acuity by about 40% (Figure 5(a)). These data demonstrate for the first time that a late-onset deprivation provokes a striking enhancement of sensory-driven behavior mediated by the spared sensory cortices. These results support the hypothesis that the deprivation of one sense rapidly refines sensory processing in the spared sensory cortex and improves sensory-guided behavior. The finding that visual cortex-mediated behavior is massively enhanced after WD indicates that this is caused by the refinement of visual processing in the spared V1 [65]. This idea is strongly supported by the finding that measurements of visual acuity and contrast sensitivity determined in the behavioral task and in V1 were practically identical, even at the level of individual animals [65]. In general, responses of systemic neuronal populations in V1, which are evoked by visual stimuli in mice, have been shown to correspond to measurements of visually guided behavior [32, 65, 68, 69] suggesting that responses at the systemic level of V1 are highly relevant for normal behavior. Collectively, these findings make it reasonable to conclude that compensatory changes in the spared sense, in response to the cross-modal deprivation of sensory inputs, are present not only at the level of primary sensory cortices but also at the level of cortex-dependent behavior.

In order to visualize the visual ability changes of a mouse after whisker deprivation (WD), we modified a picture of a mouse and our animal caretaker as it might be seen by another mouse and, in particular, by another mouse after WD (Figure 5(b)). For this, the original image was first converted to its spatial frequency domain by 2D Fourier analysis along the vertical and horizontal axis. Then, the resulting spectrogram was low-pass filtered at the mouse visual spatial frequency thresholds and reconverted into an image. Of course, this way of picture editing can only provide an anthropomorphic impression how mice see the world differently after somatosensory deprivation [65].

## 5. Cross-Modally Induced Reactivation of Cortical Plasticity

The profound improvements of vision in adult mice suggest that WD induces massive plastic changes in V1, despite the fact that experience-dependent V1 plasticity normally declines with aging [71–73]. A well-established model for the investigation of general V1 plasticity levels is the so-called ocular dominance plasticity (OD plasticity). V1 responses in many mammalian species, including rodents, are dominated by the input from the contralateral eye [72, 74, 75]. In mice, for example, monocular deprivation (MD) for a few days shifts this ocular dominance (OD) away from the closed eye [64, 72]. MD in juvenile mice during their visual critical period (postnatal days 28-32) leads to a reduction of V1 inputs through the previously closed eye, the characteristic signature of “juvenile” OD plasticity [64, 76, 77]. In young adult mice around 60 days of age, however, MD causes a potentiation of V1 responses to the input through the open eye [78–80]. However, OD plasticity shows an age-dependent decline and is completely absent in fully adult mice older than 120 days [71].

Interestingly, very recently, it has been demonstrated that WD can reactivate OD plasticity in mice of this age [81]. In this study, it was shown that 7 days of MD in WD mice led to a marked reduction of V1 responses evoked by the ipsilateral (open) eye, which is the typical feature of OD plasticity found in young adult mice. Moreover, OD plasticity could be also restored after auditory deprivation (AD, CHL) [81]. What might be a potential mechanism underlying this effect? It has been suggested that the cortical inhibitory tone, which gradually increases during aging, triggers the closing of the OD plasticity period [73, 82, 83]. In correspondence with this idea, many studies could demonstrate that interventions which reduce cortical inhibition levels and thereby alter the balance between excitation and inhibition (E/I balance) can reactivate OD plasticity [84–88]. Furthermore, OD shifts can be prevented by artificially strengthening GABAergic inhibition, indicating that the reduction of cortical inhibition is indeed the central hub to restore cortical plasticity in adults [84, 85, 89, 90]. Interestingly, a very recent study demonstrated that 7 days of WD cross-modally reduced GABA levels in the spared V1 which was accompanied by an increase in the E/I ratio, as revealed by high-performance liquid chromatography (HPLC) analysis [91]. Similarly, another group found that AD also cross-modally increases the E/I ratio in V1 by selective potentiation of thalamocortical inputs onto layer 4 pyramidal neurons without changes in the excitatory drive on parvalbumin-positive inhibitory interneurons in V1 [92]. At the first glance, these findings seem to suggest that the reduction of inhibition levels in V1 (after either WD or AD) is causal for the cross-modal restoration of OD plasticity in fully adult mice. However, increasing cortical inhibition with diazepam in WD and AD mice did not abolish cross-modally induced OD shifts [91]. On the contrary, this treatment shifted the quality of OD plasticity from “adult-like” to “juvenile-like” strongly indicating that cross-modally reactivated cortical plasticity does not simply depend on alterations in the E/I balance but also requires other, so far, unknown mechanisms [91]. To the best of our knowledge, the finding that increasing cortical inhibition changes the characteristic of OD plasticity (after WD or AD) is unique and demonstrates that cross-modally restored OD plasticity in fully adult mice is qualitatively distinct from other forms of OD chances.

There is convincing evidence that plastic changes in the OD in V1 of normal juvenile or young adult mice are mediated by long-term depression- (LTD-) and long-term potentiation- (LTP-) like mechanisms, which require the activation of the N-methyl-D-aspartate (NMDA) glutamate receptor. In contrast, NMDA receptor-dependent OD plasticity is completely absent in fully adult mice beyond 110 days of age [78–80, 82, 93–95]. We could recently demonstrate that cross-modally restored OD plasticity also requires NMDA receptor activation, as blocking this receptor with the competitive NMDA receptor antagonist CPP abolished cross-modally induced OD shifts after WD or AD [81, 91]. Moreover, in line with this finding, a recent study could show that AD reactivates thalamocortical LTP in adults, which was accompanied by a potentiated function of synaptic NMDA receptors [92]. These results underline the pivotal role of these receptors in cross-modal plasticity and demonstrate that a sensory deprivation can restore NMDA receptor function of spared primary sensory cortices in adults.

Collectively, these findings provide convincing evidence that the deprivation of a nonvisual sensory modality acts to rejuvenate the spared V1. Hence, one could argue that WD (or AD) per se sets V1 back into a plastic stage where visual experience can reshape V1 circuits and thereby enables to improve visual acuity and contrast sensitivity to compensate for the loss of whisker-dependent somatosensation, which in normal rodents provides essential information about their direct environment.

## 6. Conclusions and Outlook

Here, we briefly reviewed the findings of the literature on the cross-modal interplay of primary sensory cortices and on the effects of short-term sensory deprivation on the spared sensory cortical regions. Collectively, the described results suggest that the absence of both audition and somatosensation induces a compensatory improvement of vision. Since primary sensory cortices relay the sensory information to higher-order cortical areas, it would be interesting to investigate how cross-modal modulations as described here are transferred from primary to higher-level cortices and which effects they provoke here. Generally, the fact that the functional cross-modal interplay is present between different sensory cortices across many species raises the question of its relevance for humans or animals. Since it has been suggested that corticocortical connections mediate cross-modal effects [1, 7–9], further studies are needed to examine how silencing these connections, for example, via optogenetic inactivation, affects multimodal processing at higher-order cortices and, more importantly, sensory-guided behavior in experimental animals. The results of such experiments might reveal new insights into the general relevance of cross-modal integration for sensation and perception.

In general, investigations on cross-modal compensatory changes of a spared sensory cortex are rare, and most studies were performed in very young experimental animals. There are first hints that many fundamental mechanisms like LTP, homeostatic plasticity, and synaptic scaling are involved in mediating cross-modal enhancement of the spared senses after deprivation of another sense [50, 59, 66, 92]. However, the precise cellular and molecular mechanisms underlying this type of plasticity are largely unknown. Thus, future studies should examine possible biochemical alterations in spared primary sensory cortices in young and adult experimental animals. Neuromodulators such as serotonin might be a promising candidate as it has been already demonstrated that extracellular serotonin levels increase in the spared cortex after sensory loss and act to facilitate synaptic plasticity in juvenile and adult animals [58, 84, 96]. Furthermore, as the cortical inhibitory tone is crucial for cortical plasticity in juvenile and adult mice [82, 83, 91], the neurotransmitter GABA might also be involved in mediating cross-modal compensations, as described above [91]. Finally, it would be also of great interest to investigate whether compensatory refinements and sharpening of the remaining senses are based on the reduction of cortical activity in the deprived primary sensory cortex after the loss of its main input. If so, one could then investigate whether cross-modally induced improvements are also based on direct corticocortical connections between the interacting cortices like what was described above for cross-modal integration [7–9]. In general, not only do knowing and understanding the effects and mechanisms underlying cross-modal changes extend our knowledge on how we perceive our environment, but they might also open new avenues for the treatment of sensory loss and perhaps even for some neuropsychiatric disorders.
